# Correction: Hybridized sulfated-carboxymethyl cellulose/MWNT nanocomposite as highly selective electrochemical probe for trace detection of arsenic in real environmental samples

**DOI:** 10.1039/d3ra90076b

**Published:** 2023-08-18

**Authors:** Youssef O. Al-Ghamdi, Mahjoub Jabli, Mona H. Alhalafi, Ajahar Khan, Khalid A. Alamry

**Affiliations:** a Department of Chemistry, College of Science Al-Zulfi, Majmaah University Al-Majmaah 11952 Saudi Arabia yo.alghamdi@mu.edu.sa; b Department of Food and Nutrition, Bionanocomposite Research Center, Kyung Hee University 26 Kyungheedae-ro, Dongdaemun-gu Seoul South Korea arkhan.029@gmail.com; c Chemistry Department, Faculty of Science, King Abdulaziz University Jeddah 21589 Saudi Arabia kaalamri@kau.edu.sa

## Abstract

Correction for ‘Hybridized sulfated-carboxymethyl cellulose/MWNT nanocomposite as highly selective electrochemical probe for trace detection of arsenic in real environmental samples’ by Youssef O. Al-Ghamdi *et al.*, *RSC Adv.*, 2023, **13**, 18382–18395, https://doi.org/10.1039/D3RA03808D.

The authors regret that affiliations *b* & *c* were incorrectly shown in the original manuscript. The corrected list of affiliations is as shown below.

The Royal Society of Chemistry apologises for these errors and any consequent inconvenience to authors and readers.

## Supplementary Material

